# Hempel on the Science of Homœopathy

**Published:** 1874-07

**Authors:** 


THE SCIENCE OF HOMŒOPHATHY; or, a critical and synthetical exposition of the doctrines of the Homœopathic school. By Charles J. Hempel, M.D., etc. New York, Boericke & Tafel, 1874. Octavo, cloth, pp. 177. Price $1.75.

Our author informs us in his preface that it is his object "to endeavor to develop the idea which originally gave rise to the founding of the homœopathic school, and to establish this idea upon a basis of scientific universality and exactness." In the accomplishment of this object, we may well be excused for thinking, he has signally failed. In looking over Dr. Hempel's work we are astonished with the boldness of the author in "presenting the goddess of homœopathic truth stripped of all human wrappings and adventitious rules and substitutions." One would fain believe that even the effrontery of the strictest pharisee of the sect would pale before the ghastly spectacle of the ghost of Hahnemann thus denuded of its tinselry and trappings. We are first surprised and alarmed by the announcement that: "Homœopathy is something more than a mere art, the exercise of which may afford us a *good living* and a *position* in society"—and then quickly consoled by the added assurance: "Let us remember that the medical systems of the day are the systems of men, which *pass away* and are forgotten." Scarce are we settled down into our accustomed state of equinimity when we are again stirred up by the bold assertion that the infinitely attenuated, so-called, science "is fraught with *common sense*"*!* and makes its appeal to "the prerogative of an *unshackled reason.*"

The volume is frontispieced by a steel engraving of the venerable head and grizzled beard of the author. The manner of the argument may be inferred from a few brief quotations, *e.g.*: "I have cured numerous cases of diarrhœa"—"on the other hand, I can boast of equally striking curative effects"—"positive and even brilliant results"—"awoke perfectly restored to health"—"some very brilliant results were obtained"—"vomiting ceased and *home sickness* likewise disappeared"—"cured of a purulent and very fœtid otorrhœa, with which she had been afflicted for a year, and which was attended with agonizing pains in the head every night, by means of a single drop of the thirtieth attenuation of mercurius vivus," etc.

We have no heart for argument with the homœopaths. They have but one line of argument that is worthy of attention, and this their stubborn facts. Against this logic cannot prevail, and we content ourselves with the array of facts equally stubborn and unassailable.

Years ago we were approached by a zealous disciple of the sugar pellet persuasion in the genuine spirit of a Plymouth Rock missionary. He plied us with his undoubted facts *ad nauseam.* We mildly suggested that we could well admit his facts, provided, only, he would be equally courteous. We detailed an observation of the wonderful doings of a great magician; how he crammed ordinary tow down his throat with a dagger; how he rubbed his abdomen vigorously to set in active motion its contained machinery; how he seized between his lips and drew out from his stomach, twelve yards of red silk ribbon, and an equal quantity of white and blue and green and yellow. What a wonderful stomach, with its transmuting chemical laboratory, its bleaching and dyeing works, its delicate spindles and loom! It was a stubborn fact; we witnessed it with our own eyes; more than a hundred men in Georgia to-day, men of most undoubted veracity and social position, saw and will testify to the facts just as we related them. "Everybody knows," replied the *pellet,* "that necromancy is opposed to all the dictates of reason and common sense." "Precisely!" we rejoined, "and so does everybody know that the so-called facts of homœopathy are diametrically in opposition to all human reason and all common sense."

The work before us represents the more advanced school of homœopathy. Finding the credulity of the public becoming a little threadbare, it is discovered that "the principles and practice of homœopathy embody the fullness of all that is philosophically correct in the doctrines of old school therapeutics;" and that "any dose, large or small, is legitimate and scientifically correct which will effect a cure in the most thorough, safe and expeditious manner." Our author has observed, too, the fact that in malarial dysentery "a few sugar-coated, one-grain pills of quinine were administered, after which there was no return of the disease"—and "The patient likewise took two grains of iron in the form of a sugar-coated pill every day. After this treatment had been persistently followed for four weeks, the young lady reappeared among her friends like the picture of health." And thus it is he throws a tub to the whale, and goes on to remark, in another connection: "It is time that our allopathic brethren should know that a man may be a homœopath without abjuring common sense." Can as much be said of common honesty? We fear the "tears of grief" which our author "sheds over the blindness and fanaticism which prevent allopathic physicians from studying the writings of the homœopathic school" will prove unavailing.

Much as we detest the ignorance of old Thompsonianism, and the duplicity and nonsense of Hahnemannism, we cannot blind our eyes to the fact that they have served a useful purpose in their day as fingerboards recalling the scientific student of medicine from his occasional wanderings, and pointing into the pathway of true knowledge. This, and only this, may the champions of those isms-of-one-idea justly claim.

Nor ought we wholly to contemn these sources of knowledge, however humble or misguided they may be. Well and gratefully do we recollect the timely prompting of an old crone at the bedside of a young lady who was making great complaint of hypogastric pain: "Have you passed urine lately, Miss Blank?" "Sir?" "Do you feel an inclination to empty the bladder?" "Sir?" "Have you inclination to make water?" "I don't understand you, sir." "Doctor, ask her if she wants to *pee*," whispered the old crone. It is needless to say that the dilemma was at once relieved.

